# Oral Cavity Squamous Cell Carcinoma Xenografts Retain Complex Genotypes and Intertumor Molecular Heterogeneity

**DOI:** 10.1016/j.celrep.2018.07.058

**Published:** 2018-08-21

**Authors:** Katie M. Campbell, Tianxiang Lin, Paul Zolkind, Erica K. Barnell, Zachary L. Skidmore, Ashley E. Winkler, Jonathan H. Law, Elaine R. Mardis, Lukas D. Wartman, Douglas R. Adkins, Rebecca D. Chernock, Malachi Griffith, Ravindra Uppaluri, Obi L. Griffith

**Affiliations:** 1 McDonnell Genome Institute, Washington University School of Medicine, St. Louis, MO 63108, USA; 2 Department of Otolaryngology, Washington University School of Medicine, St. Louis, MO 63108, USA; 3 Department of Medicine, Division of Medical Oncology, Washington University School of Medicine, St. Louis, MO 63108, USA; 4 Department of Genetics, Washington University School of Medicine, St. Louis, MO 63108, USA; 5 Siteman Cancer Center, Washington University School of Medicine, St. Louis, MO 63108, USA; 6 Department of Pathology and Immunology, Washington University School of Medicine, St. Louis, MO 63108, USA; 7 Department of Surgery/Otolaryngology, Brigham and Women’s Hospital and Dana-Farber Cancer Institute, Boston, MA 02215, USA; 8 These authors contributed equally; 9 Senior author; 10 Lead Contact

## Abstract

Herein, we report an oral cavity squamous cell carcinoma (OCSCC) patient-derived xenograft (PDX) platform, with genomic annotation useful for co-clinical trial and mechanistic studies. Genomic analysis included whole-exome sequencing (WES) and transcriptome sequencing (RNA-seq) on 16 tumors and matched PDXs and additional whole-genome sequencing (WGS) on 9 of these pairs as a representative subset of a larger OCSCC PDX repository (n = 63). In 12 models with high purity, more than 90% of variants detected in the tumor were retained in the matched PDX. The genomic landscape across these PDXs reflected OCSCC molecular heterogeneity, including previously described basal, mesenchymal, and classical molecular subtypes. To demonstrate the integration of PDXs into a clinical trial framework, we show that pharmacological intervention in PDXs parallels clinical response and extends patient data. Together, these data describe a repository of OCSCC-specific PDXs and illustrate conservation of primary tumor genotypes, intratumoral heterogeneity, and co-clinical trial application.

## In Brief

Campbell et al. report the genomic fidelity of patient-derived xenograft models from oral cavity squamous cell carcinomas. These models conserve the mutation and expression profile of their matched tumors, validating their use for co-clinical trial and mechanistic studies.

## Graphical Abstract

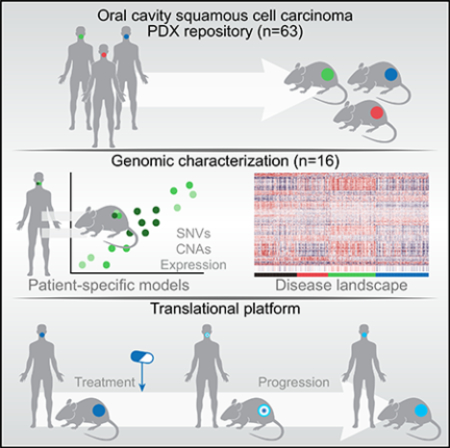

## INTRODUCTION

Oral cavity squamous cell carcinomas (OCSCCs) are a global health problem, with more than 500,000 reported cases per year. Despite major advances in surgical techniques and chemo-radiotherapy, outcomes for patients with locally advanced disease have remained unchanged at 30% local or regional disease recurrence, 25% distant metastases, and 40% overall 5-year survival (Chinn and Myers, 2015; Zhang et al., 2013). Molecular characterization using next-generation sequencing has broadened our understanding of common OCSCC genomic alterations and carcinogenesis (Cancer Genome Atlas Network, 2015; India Project Team of the International Cancer Genome Consortium, 2013; Pickering et al., 2013). Precision medicine approaches targeting specific pathways implicated in OCSCC are in early stages, with validation studies pending for a number of oncogenic dependencies (Hammerman et al., 2015). These large-scale studies would benefit from additional insight obtained using *in vivo* models that capture the complex genetic background of OCSCC.

Patient-derived xenografts (PDXs) represent a high-fidelity, personalized model for pre-clinical testing and validation of targeted therapeutics (Hammerman et al., 2015; Hidalgo et al., 2014). In addition, they provide a valuable resource for the study of intratumoral heterogeneity and clonal dynamics (Eirew et al., 2015; Hammerman et al., 2015; Hidalgo et al., 2014). A recent study of more than 1,000 diverse tumor xenografts integrated into a PDX clinical trial (PCT) framework revealed the fidelity of xenografts in confirming multiple genotype relationships with drug sensitivities (Gao et al., 2015). This study included seven PDXs derived from head and neck squamous cell carcinomas (HNSCCs). Three studies have reported initial engraftment rates for HNSCC PDXs ranging from 17%–80% but included the use of distinct immunodeficient mouse strains (Keysar et al., 2013; Li et al., 2016; Peng et al., 2013). Another study analyzed gene expression of matched primary tumors and PDXs showing variable levels of conservation, but this was limited to three cases (Guo et al., 2016; Peng et al., 2013). Interestingly, studies in larger HNSCC PDX cohorts have shown that engraftment success has no relation to pathologic stage or clinical behavior of the primary tumor (Keysar et al., 2013; Li et al., 2016). Large PDX collections are critical to capturing the population-wide genomic alterations that are obscured in analysis of smaller cohorts (Gao et al., 2015). However, existing OCSCC-specific PDX models have not been comprehensively defined to sufficiently depict the heterogeneous disease landscape.

Herein we describe a cohort of OCSCC xenografts derived from patients who have undergone standard-of-care surgery or who enrolled in neoadjuvant trametinib or pembrolizumab clinical trials. We performed sequencing analysis on 16 case-matched tumors and PDXs, which displayed genomic and transcriptomic fidelity to their respective tumors. While maintaining the mutational landscape displayed in their matched primary tumors, these PDXs also captured the molecular and genomic diversity of OCSCC at the cohort level. Our study also reports a larger OCSCC PDX repository (n = 63), which includes the subset (n = 16) with comprehensive molecular annotation reported here, that will serve as a platform for evaluating novel therapeutic approaches as well as deepen our understanding of the genomic and transcriptomic parallels between tumors and PDXs.

## RESULTS

### Generation of Xenografts

In 2013, we initiated a PDX study for OCSCC across 114 patients from three cohorts: treatment-naive primary OCSCC patients undergoing standard-of-care primary resection (n = 84), patients enrolled in a neoadjuvant trametinib clinical trial (ClinicalTrials.gov: NCT01553851; n = 20) (Uppaluri et al., 2017), and patients enrolled in a neoadjuvant pembrolizumab clinical trial (ClinicalTrials.gov: NCT02296684; n = 10, data not published). PDXs were attempted from treatment-naive tumor samples from all patients (n = 114), post-treatment surgical resections from patients enrolled in the trametinib clinical trial (n = 20) or the pembrolizumab clinical trial (n = 10), and patients with relapsed disease (n = 3). Overall, establishment of passage 0 (P0)-generation xenografts was successful in 63 tumor samples, including 45 of 114 (39.4%) treatment-naive, 10 of 20 (50%) post-trametinib treated, 5 of 10 (50%) post-pembrolizumab treated, and 3 relapse (Tables 1 and S1). PDXs were harvested once the tumor size reached 2 cm^3^, with a median time to harvest of 85 days (range, 27–281 days).

Because our goal was to develop a PDX repository for genomic and functional studies, we collected clinical and pathologic information only on samples that successfully engrafted as P0 PDXs. Demographics showed that 46 tumor specimens were from male patients and 17 tumor specimens were from female patients (Tables 1 and S1). PDXs were successfully established for 3 stage I and II, 10 stage III, and 32 stage IV primary (treatment-naive) tumors. At time of biopsy or surgery, patients were 18–87 years of age (median, 63 years). In addition, the xenograft time to harvest, when used as a measure of how aggressively the xenograft grows, was not significantly different across tumor stages. Pathologic evaluation of the PDXs was consistent with squamous cell carcinoma histology (data not shown).

### Genomic Analysis

#### Clinicopathological Summary of Sequenced Samples

We selected 16 PDXs (25% of the repository) for molecular annotation using whole-genome sequencing (WGS), whole-exome sequencing (WES), and/or transcriptome sequencing (RNA-seq). WES and RNA-seq were obtained for 16 case-matched tumors and P0 PDXs; WGS was obtained for 9 matched tumors and PDXs (Tables 1 and S1). Of these 16 xenografts, 13 were derived from primary untreated tumors, 1 was derived from a relapse tumor, and 2 were derived from a paired primary and relapse tumor. The cohort of sequenced PDXs did not significantly differ from the larger cohort of established OCSCC PDXs with regard to stage, age, and gender. There was no significant difference in the xenograft time to harvest between the sequenced PDXs and the remainder of the cohort.

#### Mouse Contamination in Xenografts

Mouse cells were not sorted from PDX samples prior to nucleic acid isolation for sequencing. Xenograft purity was defined as the percentage of sequencing reads that specifically align to the human reference genome in comparison with the mouse reference genome. Mouse contamination was highest in WGS data (9.7%–55.6% mouse-specific reads), followed by RNA-seq (5.4%–39.7%) and finally exome data (0.7%–35%), reflecting the successful enrichment of human DNA by the hybridizationbased capture reagent (Figure S1). A negligible number of reads were classified as “both,” “neither,” or “ambiguous” on the basis of the level of certainty that reads map to either human genome, mouse genome, or neither. Reads classified as “mouse specific,” “both,” “neither,” or “ambiguous” were filtered out of the sequencing data, and all subsequent analysis was performed on the reads classified as “human specific.”

#### Sequencing Results

WGS median sequence coverage was 263 for PDXs, 753 for tumors, and 373 for normal samples. WES resulted in at least 203 coverage over an average of 91.5% of the targeted exome in PDXs, 96.6% in tumors, and 95.2% in normal samples and an average depth of 82.43 in PDXs, 81.23 in tumors, and 66.23 in normal samples. The total number of reads generated by RNA-seq ranged from 89.5 million to 767 million, with an average of 391 million reads in tumor samples and 226 million reads in PDX samples. Metrics for PDXs correspond to human-specific reads, after competitive alignment with the mouse genome.

### The Landscape of Somatic Mutations Is Conserved in Most OCSCC PDXs

**Figure 1.**
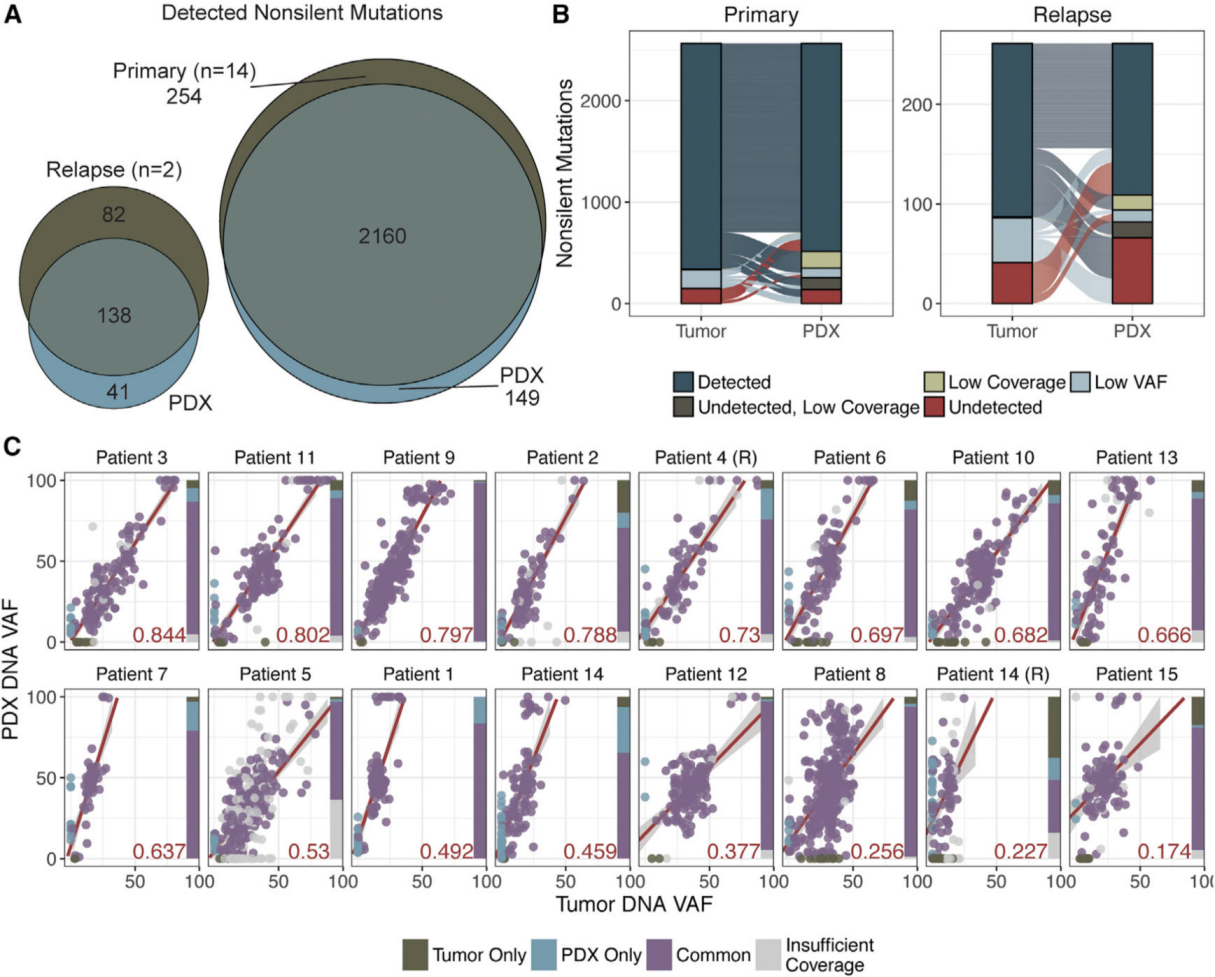
Mutations Are Overall Conserved in PDXs (A) Venn diagram of all variants detected in primary or relapse tumors and their respective PDXs. (B) Alluvial plots displaying variants detected in either “tumor,” PDX, or “both.” Variants are labeled “detected” if they have sufficient sequencing depth (203) and VAF (5%); “low coverage” or “low VAF” if they are detected but do not meet one of these filters; “undetected, low coverage” if they are undetected and have insufficient coverage; and “undetected” if the variant is undetected at a position with sufficient coverage. (C) Scatterplots displaying the correlation between PDX DNA VAF and tumor DNA VAF. Samples are designated “R,” corresponding to “relapse” samples.Points are colored on the basis of which samples the variant was detected in; gray points indicate variants for which there was <203 coverage in either the tumor or PDX sample. The R^2^ value (of common points with at least 203 coverage in both samples) is represented by the red value in the lower right-hand corner of each plot. The linear regression line is indicated in red with boundaries showing the SD of points. The bar charts on the right of each plot indicate the proportion of common versus sample-specific variants, as well as those with less than 203 coverage in either the tumor or matched xenograft sample (indicated in gray). Refer to Figure S2 and Table S2 for further details.

Somatic alterations detected by WGS, WES, and/or RNA-seq were compared for the 16 pairs of OCSCC PDXs and case-matched tumors. There were 2,414 non-silent coding single-nucleotide variants (SNVs) and small insertions and deletions (indels) detected in 14 primary tumors (Table S2; Figure S2A). Of these, 1,929 (79.9%) were also identified in matched PDXs with sufficient coverage (203) and variant allele fraction (VAF; 5%). Our somatic validation pipeline subjects variant calling to additional filtering on the basis of sequencing coverage and read support. Variants were identified independently in tumors and PDXs, and then the union of these events was re-analyzed in both samples to detect and recover variants with low sequencing coverage and/or VAF. An additional 231 variants (9.6%) originally detected in primary tumors were accounted for in the PDXs. Overall, 89.5% of all variants identified in primary tumors were also detected in their matched PDXs (Figures 1A and 1B). In the 2 relapse tumors, 220 non-silent SNVs and indels were identified; however, 119 (54.1%) were confirmed in matched PDXs, and only 19 (8.6%) were recovered by reducing sequencing depth and VAF filters. Overall, only 62.7% of variants detected in relapse tumors and their matched PDXs were shared (Figures 1A and 1B).

PDXs were compared with their respective tumors on the basis of the percentage of tumor variants maintained in their respective PDXs and linear regression across the VAF distributions (Figure 1C). This analysis was restricted to variants that had at least 203 coverage in both tumor and PDX samples. Twelve PDXs (75%) retained at least 90% of the variants detected in their respective tumors. Two of the remaining four tumors (patients 2 and 6 primaries) had relatively higher correlation in VAF distribution (R^2^ = 0.788 and 0.697, respectively) of shared variants, the other two (patient 15 primary, patient 14 relapse) had the lowest correlation coefficients (R^2^ = 0.174 and 0.227, respectively) but also had much lower tumor cellularity in the tumor sample (less than 50%). Overall, the correlation in VAF distribution was lower in relapse cases (0.23–0.73; median, 0.48; n = 2) than primary cases (0.17–0.84; median, 0.65; n = 14). However, these aggregate metrics are reduced because of the cellularity of patient 14’s relapse tumor, which had the lowest tumor purity (~25%).

We next evaluated our PDX cohort for conservation of variants in previously described significantly mutated genes for the HNSCC TCGA (The Cancer Genome Atlas) cohort (Cancer Genome Atlas Network, 2015). There were 47 mutations identified across 12 of the genes described as significantly mutated genes from the TCGA HNSCC cohort (Cancer Genome Atlas Network, 2015), and 44 were detected in both tumors and PDXs across 16 tumors (1–5 mutations per patient; median, 2). The reported cohort included mutations in *TP53* (n = 12 primary, n = 2 relapse), *NOTCH1* (n = 3 primary), *KMT2D* (n = 2 primary), *HRAS* (n = 2 primary), *FAT1* (n = 2 primary), *CDKN2A* (n = 4 primary, n = 2 relapse), *CASP8* (n = 3 primary, n = 1 relapse). Mutations were also detected in *AJUBA, CUL3, FBXW7, NSD1,* and *PIK3CA*, each in only one primary sample. In 15 of these tumors, all putative driver mutations were preserved, while indels in *FAT1* were observed in either the relapse tumor or PDX from patient 14. Despite variance in the correlation coefficient across the cohort, putative drivers that have been previously described in HNSCC were maintained (Table S2; Figure S2).

### OCSCC PDXs Do Not Exhibit Rapid Accumulation of Mutations Post-engraftment

In order to evaluate whether mutations could have been acquired after engraftment, we identified variants in PDXs that were undetectable in the primary tumors. There were 149 PDX-specific variants across the 14 primary PDXs, 76 (51%) of which were expressed in the RNA; there were 41 PDX-specific variants detected in 2 relapse PDXs, 20 (48.8%) of which were expressed in the RNA (Figure 1B). Of the 190 variants exclusively detected in PDXs, 4 (2.1%) had 0–20× coverage in their respective tumors, but the coverage of the genomic positions of PDX-specific variants in tumors (0–672×; median, 164×) was not significantly different from the coverage of these variants in PDXs (20–525×; median, 88.53). Thirty-seven of the 190 variants (19.5%) were detected at ≤5% VAF in the PDX. However, to consider whether PDX-only mutations were acquired post-engraftment, we evaluated the clinical significance and potential implication in tumorigenesis. Of the 190 PDX-only variants, only 2 were described as recurrently mutated in the TCGA cohort. Both variants were frameshift indels in *FAT1* in the patient 14 relapse PDXs, present at 53% and 61% VAF, respectively. Only one of these was expressed at the RNA level (32.3% VAF). It is important to note that the patient 14 relapse tumor had the lowest purity (~25%) and had the second lowest correlation coefficient in VAF distribution with its respective PDX.

Studies evaluating the genomic integrity of PDX models across tumor types have described the selective pressure and/or accumulation of mutations over several passages. In order to address this question of selective engraftment and pressure to acquire mutations via passaging, the parental xenograft (P0) generated from patient 13 was passaged twice in NSG mice. Three PDXs from the P2 generation were studied by WES. Of the 104 variants detected in the primary tumor, 90 (86%) were detected in the parental PDX. Out of the 90 variants confirmed in P0, 85–87 (94%–97%) were subsequently detected in the P2 generation PDXs (Table S2). There were 7 variants detected in the P0 PDX that were undetectable in the primary tumor, 6 of which were also detected in all three P2 PDXs. There were 11 variants detected in P2 PDXs that were not detected in either the primary tumor or the P0 PDX; 5 of these variants were present in all P2 PDXs (VAF = 4.44%–27.78%). Six of these variants were specific to one or two of the P2 PDXs, all present at less than 10% VAF. However, overall, correlation across all variants remained 0.85–0.88 between all PDXs and the primary tumor.

### Large and Focal Copy-Number Alterations Are Retained upon Engraftment

**Figure 2.**
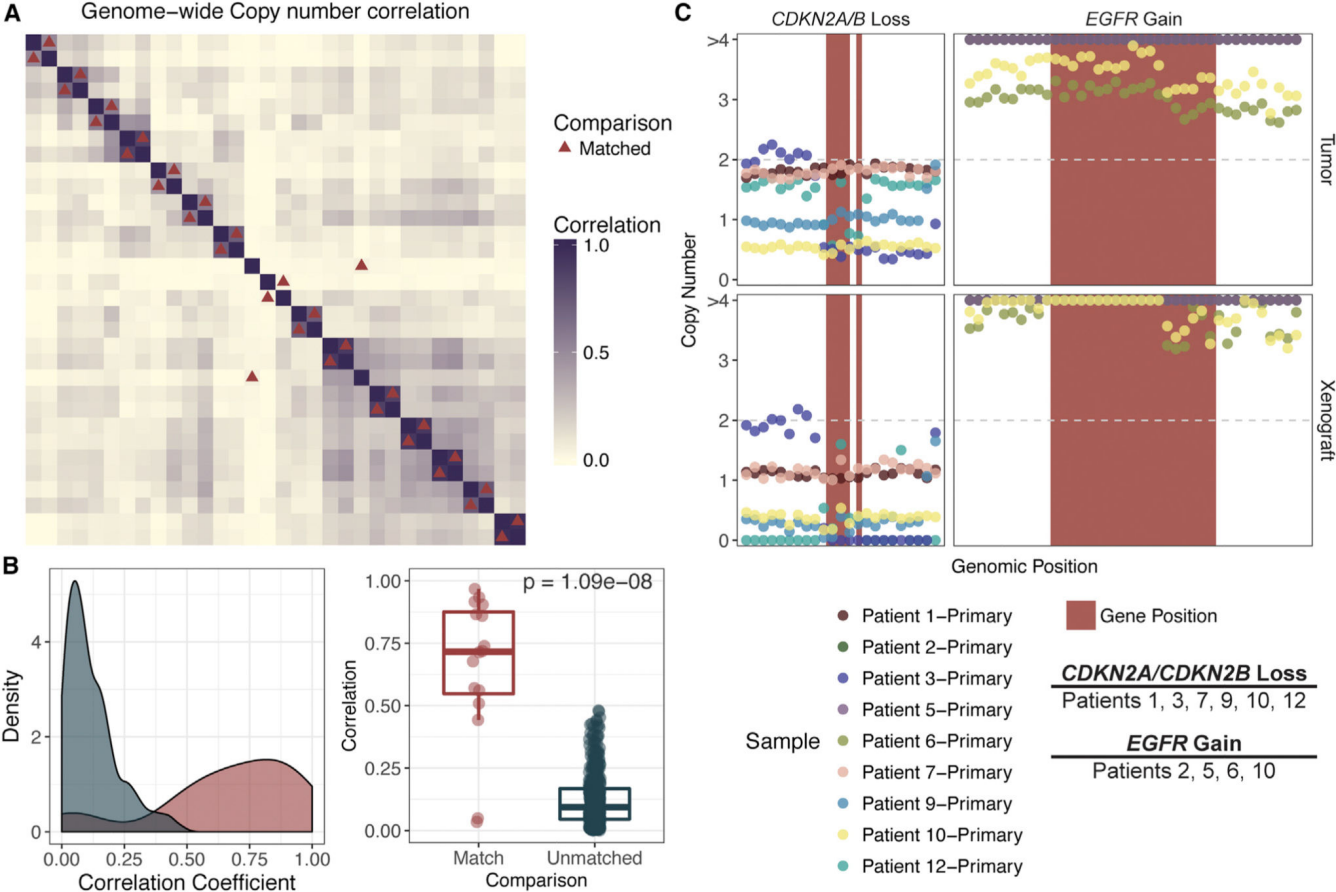
Copy-Number Alterations Are Concordant in Matched PDXs and Tumors (A) Correlation matrix displaying the Pearson correlation coefficient (calculated on the basis of the absolute copy-number segment mean across 10 kb windows). Samples are sorted on the basis of unsupervised hierarchical clustering of the correlation coefficient. Red triangles correspond to matched tumors and PDXs. (B). Density plot showing differences in correlation coefficient between case-matched tumors and PDXs (red) versus any other comparison (blue). A Wilcoxon test was performed, comparing the correlation between case-matched PDXs and unmatched or distinct pairs of samples (p = 1.09e-08). (C) Genes commonly altered at the copy-number level in HNSCC were analyzed with 100 kb windows on either ends of the gene. Red rectangles correspond to the genomic positions of the indicated gene. Point color corresponds to sample. Copy number is indicated by absolute copy number on the y axis, and only segments with median copy number > 3 or < 1.5 are indicated by color (according to sample source). Refer to Figure S2 for further details.

Absolute copy number was calculated by comparing either tumor or PDX data with matched normal data and evaluated on the basis of 10 kb windows across the genome. In order to evaluate whether copy-number alterations (CNAs) were conserved at the genome-wide scale, we calculated the correlation between all case-matched PDXs and tumors. The Pearson correlation coefficient between matched tumors and PDXs ranged from 0.3 to 0.97 (median, 0.72; median, 0.08 for unmatched samples; Figure 2A). We found that correlation between samples was significantly higher in matched PDXs and tumors than in comparison with any other pair of samples (p = 2.88e-07; Figure 2B). There were six samples that had relatively low Pearson correlation coefficients (r < 0.60). Of these six samples, one (patient 14, relapse, r = 0.561) had low tumor purity (25%). Two (patients 5 and 8) had very low correlation coefficients (r = 0.034 and r = 0.049, respectively). Interestingly, these two patients also had the highest mutational burdens (n = 445 and n = 327, respectively). Lack of correlation might again be attributable to lower cellularity and/or cases with large numbers of somatic alterations possibly indicative of increased genomic instability.

Recurrent CNAs included gains in chromosomes 8q (n = 7), 5p (n = 5), and 3q (n = 4) and losses in chromosomes 8p (n = 6) and 3p (n = 5) (Figure S2B), consistent with previous studies (Cancer Genome Atlas Network, 2015). We also evaluated genes known to be contained in focal CNAs (Figures 2C and S2C). We detected amplifications of CCND1 (n = 7), EGFR (n = 4), FGFR1 (n = 1), KRAS (n = 2), and PIK3CA (n = 2) and loss of CDKN2A/ CDKN2B (n = 6) in tumors and their respective PDXs. In most cases, CNAs (segment mean > 3 for amplifications, segment mean < 1.5 for loss) were detected in both tumor and PDX. However, in a few cases, resolution of these copy-number changes was not obtained in tumors, because of low purity, but was detected in the PDX; for example, *KRAS* amplification in patient 1 (Figure S2C) and *CDKN2A/B* loss in patients 1 and 7 (Figure 2C).

### RNA-Seq Analysis Reveals Tumor-Infiltrating Cell Populations

**Figure 3.**
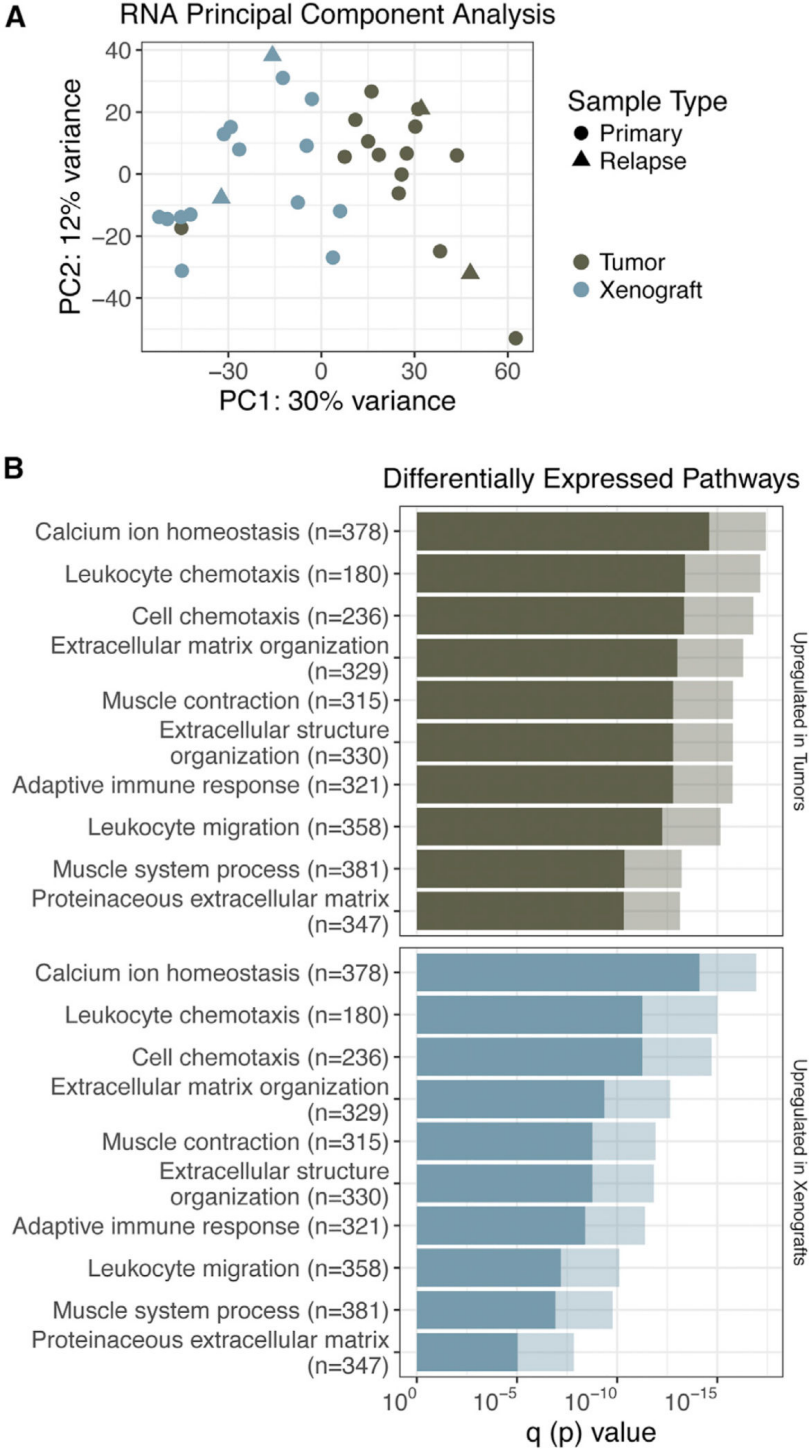
Differential Expression Analysis Reveals Tumor-Infiltrating Cell Populations (A) PCA clustering of PDX (xenome-filtered) and primary tumor RNA samples. (B) Pathway analysis is summarized by bar charts showing the p value (lighter hue) and false discovery rate (FDR) q value (darker hue). Pathways are labeled along the y axis; the number of genes annotated within each pathway is indicated in parentheses. Refer to Table S3 for further details.

Mouse-specific reads were filtered *in silico* from the xenograft RNA samples before aligning reads to the human genome. Principal-component analysis (PCA) of matched tumor and xenograft gene expression revealed distinct stratification of PDXs and tumor samples (Figure 3A). Using a supervised analysis comparing matched tumors and PDXs, there were 298 Kyoto Encyclopedia of Genes and Genomes (KEGG) pathways and Gene Ontology (GO) annotations that were significantly upregulated in tumor samples (p < 0.001), 28 processes that were significantly downregulated in tumor samples (p < 0.001), and 14 KEGG pathways that were differentially regulated within tumor and PDX samples (p < 0.001; Table S3). The top 10 most significantly upregulated processes in tumor samples consisted of cellular processes specific to nontumor infiltrating cells, such as “leukocyte migration,” “adaptive immune response,” and “leukocyte chemotaxis” (Figure 3B). Pathways upregulated in PDXs included those related to keratinization and epidermal cell differentiation.

**Figure 4.**
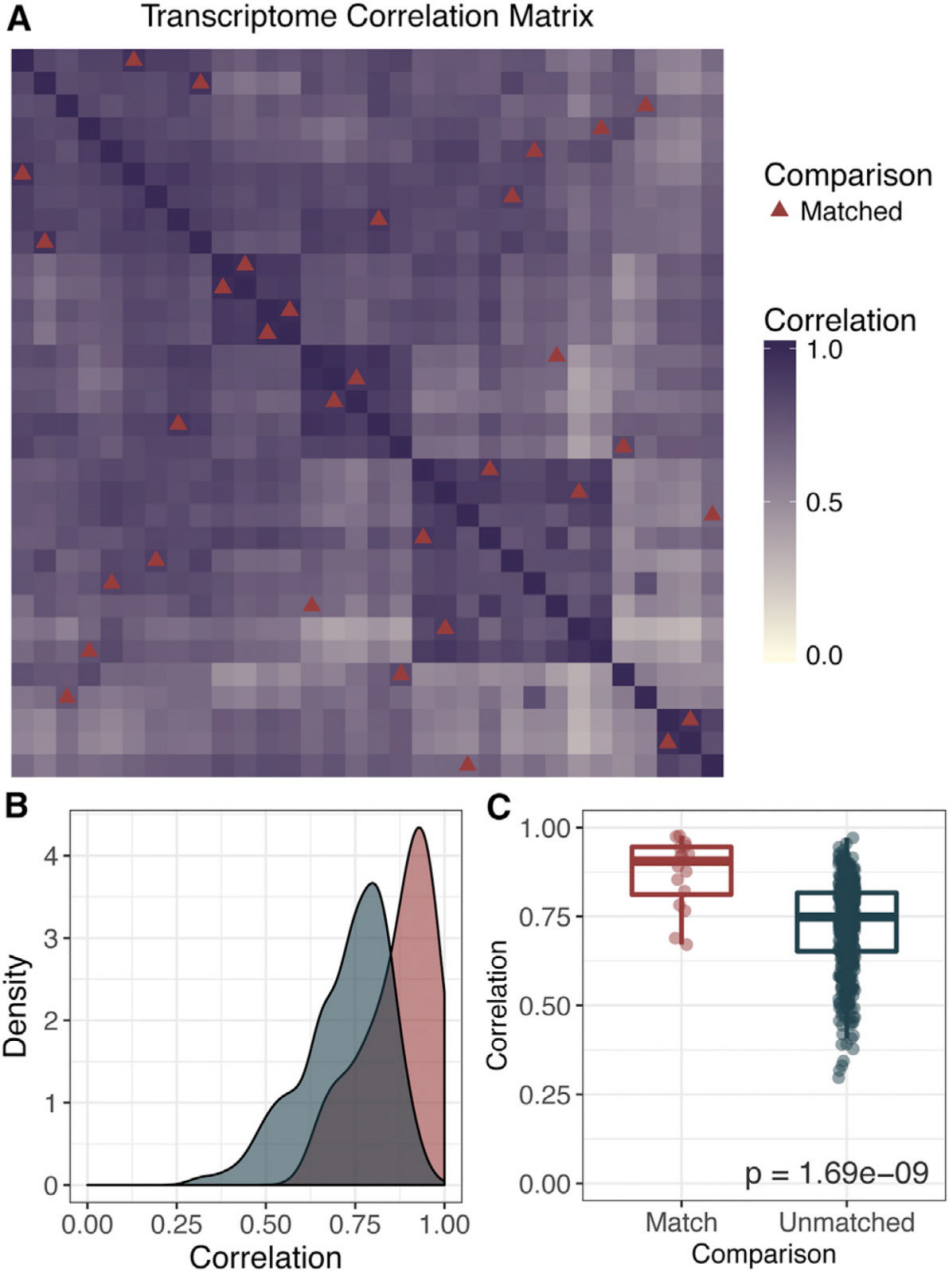
Correlation across the Transcriptome Is Highest in Matched Tumors and PDXs (A) Correlation matrix displaying the Pearson correlation coefficient calculated across the gene expression of 59,884 genes (FPKM). This included the whole transcriptome with the exception of the top 1% of genes contributing to the principal components in Figure 3. Red triangles indicate tiles corresponding to case-matched tumors and PDXs. (B) Density plot showing differences in correlation coefficient between case-matched tumors and PDXs (red) versus any other comparison (blue). (C) A Wilcoxon test was used to compare the correlation between case-matched PDXs and unmatched or distinct pairs of samples.

Independent of infiltrating cell populations, we predicted that PDXs would behave most similarly to their matched tumors compared with unmatched tumors. In order to address this question, we removed the top 1% of genes contributing to each principal component from the previous analysis. This removed the most prevalent genes associated with infiltrating cell populations in order to better evaluate genes associated with tumor-intrinsic biology (n = 59,884 genes). Pearson correlation coefficients ranged from 0.47 to 0.97 (median, 0.87) for case-matched tumors and PDXs (Figure 4A). This was significantly greater than the correlations drawn between unmatched combinations of samples (0.29–0.97; median, 0.75; p = 1.69e-09; Figures 4B and 4C).

### PDXs Recapitulate the Molecular Heterogeneity of the Disease

Previous studies have described diverse molecular subtypes in HNSCC (Chung et al., 2004; Walter et al., 2013). The four HNSCC molecular subtypes described by Walter et al. (2013) were subsequently confirmed in the TCGA dataset—atypical (24%), basal (31%), classical (18%), and mesenchymal (27%)—on the basis of genes associated with each signature (Cancer Genome Atlas Network, 2015). Because of the genomic diversity observed in our sequenced PDX cohort, we hypothesized that our repository contained PDXs derived from tumors across these molecular subtypes. To test this, we built a random forest classifier to categorize our samples on the basis of the expression signatures and previously reported molecular subtypes described in the cohort of Walter et al. (2013) (Experimental Procedures).

**Figure 5.**
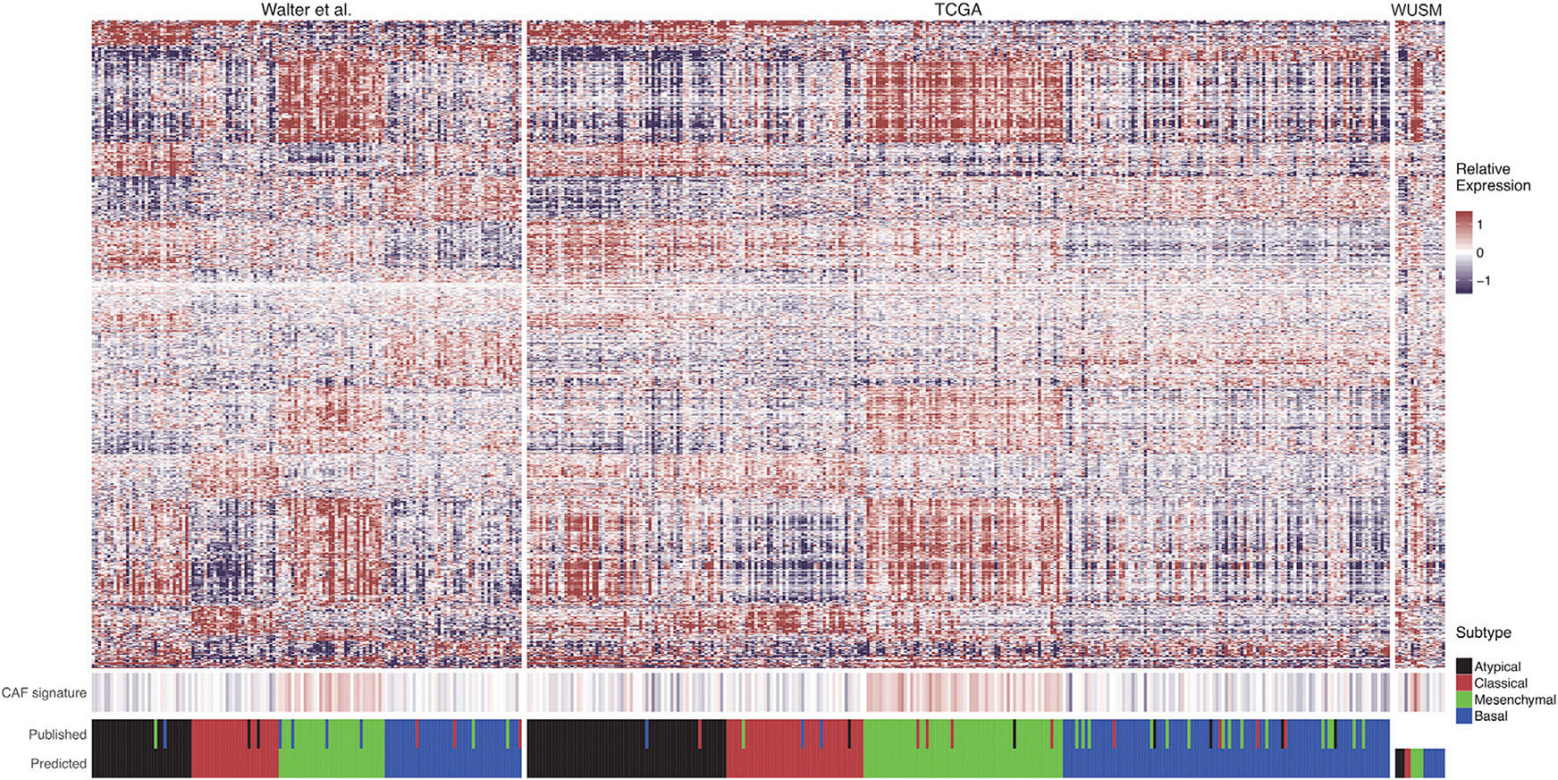
Varied HNSCC Molecular Subtypes Successfully Engraft as PDXs This heatmap contains genes (rows) corresponding to a single gene out of the 638 gene signature defining the four molecular subtypes in HNSCC. Each column corresponds to a sample within each cohort. Fill color represents the gene median-centered (GMC) value of the respective gene expression within each dataset (relative expression). Four hundred twelve genes associated with cancer-associated fibroblasts (CAFs) defined by Puram et al. (2017) were summarized by the median GMC value of the 412 genes in the associated sample (denoted in CAF signature). Datasets shown include the Walter et al. (2013) dataset (used to build the classifier), the TCGA dataset, and the 16 tumor RNA samples obtained at Washington University School of Medicine reported in this study (WUSM). Values in “predict” indicate the molecular subtype predicted by the random forest classifier described. Values in “published” indicate the molecular subtype documented for each corresponding sample in the previously published datasets. Refer to Table S4.

Our classifier, built on 638 genes, successfully categorized 125 of 138 samples in the dataset of Walter et al. (2013), for an overall accuracy of 90.6% (Table S4). When applied to the TCGA HNSCC dataset, 243 of 277 samples (87.7%) were correctly predicted within their previously published molecular subtype, accurately classifying 62 of 68 atypical, 82 of 85 basal, 40 of 49 classical, and 59 of 75 mesenchymal tumors (Figure 5). The most common incorrect classification involved identifying previously reported mesenchymal tumors as basal (15 of 75). When applied to the 16 OCSCC tumors in our study, 3 were predicted to be atypical (18.8%), 7 basal (43.8%), 2 classical (12.5%), and 4 mesenchymal (25%) (Table S4). Recently, HNSCC single-cell RNA sequencing characterized the “mesenchymal” expression signature as driven primarily by stromal infiltrate (Puram et al., 2017). Two subpopulations of cancer-associated fibroblasts (CAFs) contributed specific signatures, and we evaluated whether the CAF gene signature was upregulated in samples subtyped as “mesenchymal” from our classifier. CAF genes (n = 412) were queried across the published datasets (Walter et al., 2013, and TCGA) and our reported dataset (WUSM). Samples classified in the mesenchymal subtype displayed significantly increased relative expression in CAF-associated genes compared with the atypical (p < 0.05 in all datasets), basal (p < 0.05 in all datasets), and classical subtypes (p < 0.01 in the Walter et al., 2013, and TCGA datasets).

### PDX Parallels Clinical Response in a Trametinib “Co-clinical” Trial

Advantages of generating PDXs in coordination with clinical trials include the ability to functionally dissect patient treatment responses, as well as discovering and validating therapeutic mechanisms. Twenty-nine of the PDXs in this study were from patients in clinical trials, 10 of which are included in the sequenced cohort. As a validation of this approach and this PDX repository, we evaluated the efficacy of trametinib in the post-treatment PDX generated from patient 2 at the time of surgical resection. This patient had a subjective clinical response with a downstaging of tumor from a clinical T3N1M0 OCSCC to pathologically staged T1N0 disease (Uppaluri et al., 2017). The patient later developed a local recurrence and lung metastasis and ultimately succumbed to this disease. We analyzed WES and RNA-seq data from untreated and post-trametinib treated PDX and primary tumor samples and WES from recurrence and lung metastasis biopsies.

**Figure 6.**
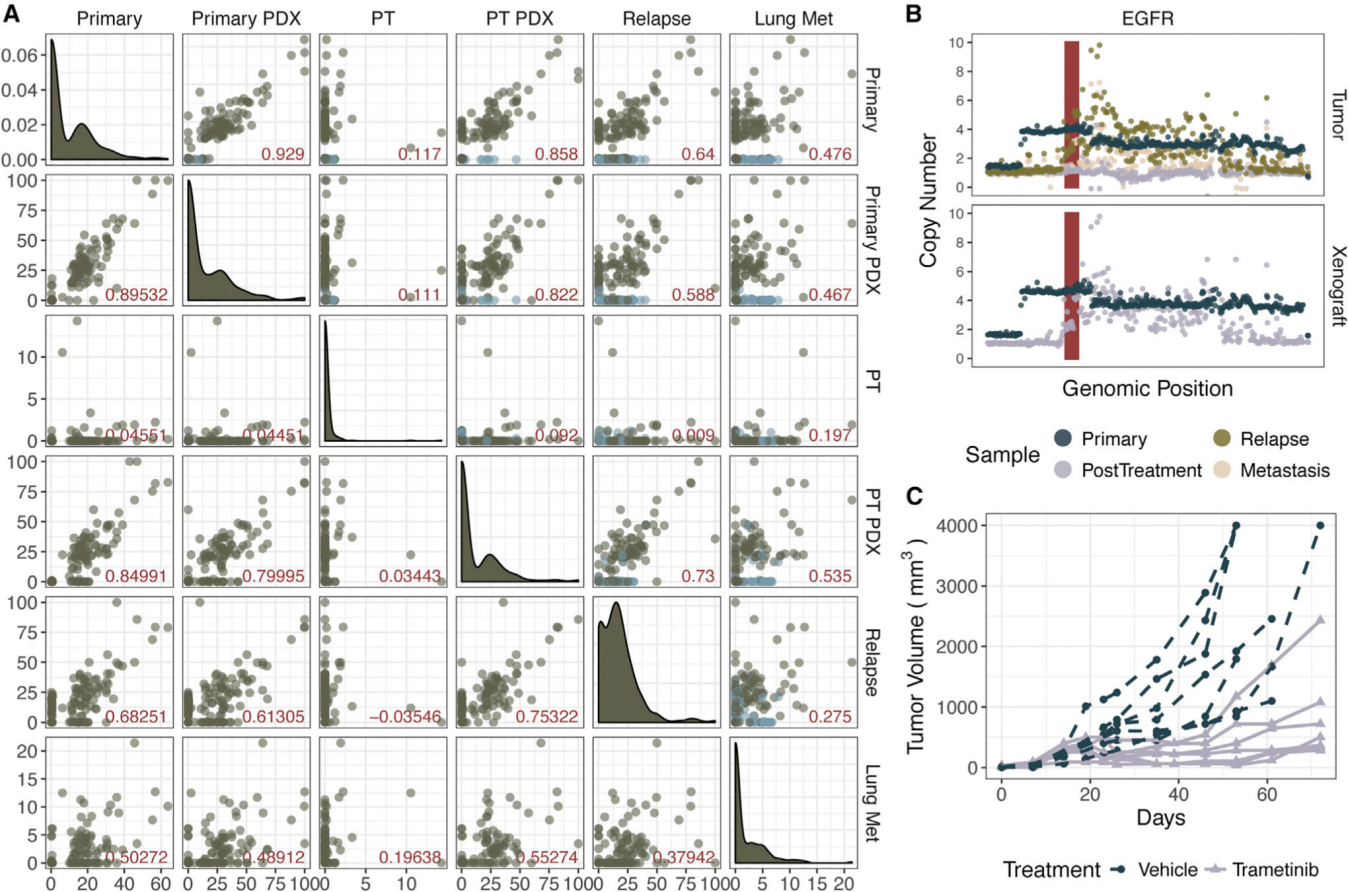
Patient 2 PDX Parallels Clinical Response in a Trametinib “Co-clinical” Trial (A) Variant allele frequency (VAF) of the y axis labels (right-hand side) and the x axis labels (across top) are indicated by each point. The upper triangle contains all variants, either detected in the primary tumor (dark blue) or detected in a subsequent sample (green). The lower triangle contains only variants detected in the primary tumor. Pearson correlation coefficient is indicated by the red value in the lower right-hand corner of each plot. Density plots along the diagonal indicate the VAF density in the corresponding sample. (B) Absolute copy number is plotted along the y axis. Each point corresponds to the segment mean calculated across 10 kb (per sample) windows within the shown genomic coordinates. The *EGFR* locus is shown in red. (C) Tumor growth comparison between vehicle-treated and trametinib-treated P2 xenografts.

The treatment-naive tumor biopsy and matched PDX have been presented in this study along with the other primary tumors. WES detected 123 SNVs and indels in the primary tumor, 92 of which were detected in its derived xenograft (Figure 6A). The post-treatment tumor sample had very low purity; only 20 of the 123 variants detected in the primary, untreated tumor were detected at less than 15% VAF (Table S2). However, in the PDX corresponding to the post-treatment tumor sample, 83 variants (67.5%) from the primary tumor were detected, as well as 14 new variants. A focal amplification of *EGFR* was observed in the primary tumor, pre- and post-treatment PDXs, the recurrent tumor, and, despite low purity, the metastasis sample (Figure 6B).

Because there appeared to be a clinical response in the 2 week “window” clinical trial, we asked whether a longer course of trametinib would result in clinical benefit using the PDX model. Cohorts of mice engrafted with patient 2’s post-treatment PDX were treated with an extended course of trametinib or vehicle (n = 7 each). While the vehicle-treated mice showed progressive tumor growth, trametinib treatment resulted in reduction in tumor size over the first 50 days of treatment, followed by outgrowth of all tumors (Figure 6C). Thus, this PDX model displays responses consistent with the clinical findings in the patient and illustrates that escape tumors can be further studied to define the basis of response and resistance.

## DISCUSSION

The PDX cohort in this study was focused exclusively on OCSCC patients, capturing clinical, mutational, and gene expression subtyping of the disease defined by molecular annotation of 25% of the available repository. Future studies will involve further genomic and molecular annotation of the remaining repository (n = 47), with appropriate public accessibility to these data. By comparing the genomic landscape of our PDX cohort to previous studies, we show that we have successfully generated diverse genotypes that span the phenotypic heterogeneity characteristic of HNSCC. Known recurrently mutated genes (e.g., *TP53, CASP8, CDKN2A*) and CNAs of chromosomes 3, 5, and 8 were recurrently altered in our sequenced cohort and were confirmed in their matched PDXs. In addition, driver events, including canonical hotspot mutations and amplifications of known oncogenes (e.g., *HRAS, PIK3CA*) and inactivation and loss of tumor suppressor genes, were confirmed in matched PDXs.

Previous studies have concluded that effective xenoengraftment does not correlate with patient age or tumor stage, while others have described the difficulty in engraftment of tumors from patients with early-stage disease. We successfully established PDXs from five patients with stage I or II disease (8% of our overall cohort). However, because our focus was on developing a repository and we studied only successfully engrafted tumors, we cannot comment on overall correlations of staging and engraftment success.

In comparing our sequenced cohort with the previously described molecular subtypes of the disease, our PDXs were shown to be established from atypical (n = 3), basal (n = 7), classical (n = 2), and mesenchymal (n = 4) tumors. The molecular subtype classifier in this study was trained and validated on previously published tumor expression data and likely includes tumor-infiltrating cell populations and microenvironmental factors of those tissues. We attempted to apply the classifier to the PDX RNA samples in addition to the tumor RNA samples, but only seven PDX samples (44%) were labeled with the same classification as their matched tumor. The random forest classifier labels samples by assigning a probability that a sample fits into a subtype, and these values were more marginal in PDX samples than in the tumor RNA samples. Discordant labels could also reflect issues in tumor purity. Future studies are necessary to describe how these molecular subtype classifications can be applied (or re-trained) to appropriately stratify large PDX cohorts. Recent studies using single-cell transcriptomics (scRNA-seq) have shown that the mesenchymal subtype of HNSCC is due primarily to infiltrating stromal cells, specifically CAFs (Puram et al., 2017). Without assessing our tumor samples at single-cell resolution, we cannot definitively attribute the mesenchymal signature in our classifier to non-tumor cell populations. However, we did observe significantly increased expression in these genes in samples classified as “mesenchymal” compared with other molecular subtypes. Future studies would benefit from additional scRNA-seq experiments to improve molecular subtyping of tumor-intrinsic patterns in HNSCC, while accounting for stromal infiltration.

Although our sequenced cohort captures genomic alterations at the population level, this study also showed how effectively OCSCC PDXs individually recapitulate their respective tumors. This establishes the potential utility of our repository to explore mechanisms of targeted drug sensitivity and resistance for precision oncology applications. Concordance was described in terms of the maintenance of mutations and genomic alterations in PDXs. At the individual level, most xenografts clearly displayed strong conservation of these alterations with their matched tumors. Previous studies have described selective environmental pressures in PDXs across tumor types, observing subclonal outgrowth or the selective engraftment of a subpopulation of cells. However, in this study comprising PDXs specifically from the P0 generation, 89.5% of all variants detected in primary tumors were retained in their matched PDXs.

There were four PDXs that did not retain at least 90% of the variants detected in their respective tumors. However, two of these still had relatively high correlation in VAF distribution of shared variants (R^2^ = 0.697–0.788), and the other two tumors had low tumor cellularity, which led to lower correlation coefficients in paired samples. Technical and biological contributions to lower correlation include sampling noise in sequencing data, causing higher variance in the VAF distribution; tumor purity, reducing the sensitivity for detecting somatic mutations; and increased mutational burden, indicative of carcinogen-induced tumors and genomic instability and resulting in increased subclonal and private mutations. It is possible that a more complex subclonal architecture could be resolved with increased sequencing depth, single-cell sequencing analysis, or further passaging to evaluate for subclonal selection. These additional experiments would account for differential engraftment of tumor cell subpopulations. Importantly, even in PDX-primary pairs with low correlation metrics, all mutations in putative driver genes were retained in the corresponding PDX in 15 cases.

The challenges contributing to low correlation metrics in mutational frequency (i.e., tumor purity, increased genomic instability, and lack of resolution of clonality) apply to copy-number detection as well. We observed concordance (Pearson correlation coefficient = 0.47–0.97) in 13 cases and very low correlation values (0.02–0.05) for 3 samples. Two of these samples, patients 5 and 8, were the most highly mutated and displayed increased genomic instability. Single-cell resolution approaches may clarify whether CNAs created a selective advantage for engraftment.

Many PDX-specific mutations (55.3%) were missed in the tumor because of low sequencing coverage or were present at lower than 5% VAF in the xenograft. This indicates that either there was not enough coverage to identify the variant in the tumor, or it may have been acquired in a very small number of cells after engraftment. In addition, we evaluated WES from three P2 xenografts derived from one of our P0 xenografts, and only 11 total mutations were detected specifically in P2 xenografts, 5 of which were present at similar frequencies across the three P2 xenografts, suggesting that they may have been selected within the P1 generation. Additional studies of a larger cohort of later passage PDXs is needed to confidently evaluate whether OCSCC PDXs generally retain the primary tumors’ genomic landscape through passages. However, our dataset does not overall exhibit aggressive mutational accumulation in early passages. In 15 of 16 cases, mutations in the all reported recurrently mutated genes were maintained. However, in the patient 14 relapse tumor, there were two mutations in *FAT1* in the PDX and a tumor-specific *FAT1* mutation. It would require more tumor sample or deeper sequencing in order to identify whether this mutation was present at lower frequencies in the tumor, because the purity of this tumor was about 25%. Although we see concordance across drivers, this example emphasizes the known fact that selective pressure in the mice does sometimes fundamentally alter tumor biology, and identifying these underlying differences is important when using PDXs.

As expected, unsupervised approaches to gene expression analysis revealed the presence of non-tumor cells in bulk primary tumor RNA data. Supervised differential expression analysis directly comparing tumors with PDXs validated this observation, revealing the upregulation of cellular processes associated with non-tumor cells (e.g., leukocyte migration, and adaptive immune response). Downregulated pathways in tumors, on the other hand, included cellular processes such as keratinization and epidermal cell differentiation. This is likely indicative of tumor purity and non-tumor cell infiltration, because PDXs represent a purer tumor cell population derived from squamous cell carcinoma tissue. When genes associated with these cellular processes were removed, we found that PDXs behaved most similarly to their matched tumors. This is consistent with other studies focused on characterizing HNSCC PDXs, using proteomics and immunohistochemistry, that show conservation of oncogenic pathway activation and biomarker expression (Keysar et al., 2013; Li et al., 2016).

This study presents the advantages of PDX models as a biological and translational platform for studying OCSCC. At the disease level, we see that the known molecular heterogeneity is captured within the described PDX cohort. More importantly, however, we validate the use of these models in a patientspecific context, demonstrating strong concordance between PDX and primary tumors and conservation of key putative driver events. We describe a patient (patient 2) who responded to trametinib in a neoadjuvant window clinical trial and show that treatment of the patient’s PDX with trametinib demonstrates a significant response closely reflecting the clinical history. For this reason, we emphasize not only the PCT framework but also the integration of PDXs into a co-clinical trial approach. The time frame required to generate PDXs does not make it feasible to study a PDX during the course of patient diagnosis and treatment. However, by generating PDXs in conjunction with patients enrolled in these neoadjuvant trials, we can compare the course of tumorigenesis with clinical outcomes and retrospectively study mechanisms of drug response. Our repository contains PDXs derived from tumor samples at various stages and time points in disease, including 29 from patients enrolled in clinical trials. Future studies will further demonstrate utility of our PDX platform as a resource for biomarker discovery, novel combinations, and targeted therapies, as well as implementation for mechanistic studies.

## EXPERIMENTAL PROCEDURES

### Sample Acquisition

The tumor acquisition protocol, clinical trials, and correlative studies were all approved by the Washington University Human Research Protection Office and Animal Studies Committee, respectively. After informed consent, samples were obtained through two methods: (1) OCSCC patients undergoing surgical biopsy or resection were recruited as part of the Washington University tumor banking protocol (institutional review board [IRB]: 201102323), or (2) patients were recruited for neoadjuvant clinical trials with either the MEK inhibitor trametinib (ClinicalTrials.gov: NCT01553851; IRB: 201205124) (Uppaluri et al., 2017) or pembrolizumab (ClinicalTrials.gov: NCT02296684; IRB: 201412118).

### Xenoengraftment Procedures

Tumor biopsies were obtained from patients and maintained in sterile DMEM containing 10% fetal calf serum (FCS) and 1% amphotericin. Biopsies were sectioned using razor blades into four separate pieces, one specifically for xenograft generation. Briefly, fresh tumor was minced into approximately 16 pieces, ranging from 2 to 8 mm^3^, and transferred on ice to the animal facility. Six- to 8-week-old NOD-*scid* ILRg^null^ (NSG) mice (The Jackson Laboratory) were anesthetized and shaved, and four small incisions were made, one on each quadrant of the flank. Tumor pieces were then saturated with Matrigel (Corning), and four pieces were transferred subcutaneously into each quadrant using sterile forceps. See Supplemental Experimental Procedures for further details on xenoengraftment, mouse maintenance, and treatment.

### Sequencing and Data Analysis

Genomic DNA was isolated by the Siteman Cancer Center Tissue Processing Core using the DNeasy Blood and Tissue Kit (QIAGEN). Library construction and sequencing were performed as previously described, with a few exceptions described in the Supplemental Experimental Procedures (Griffith et al., 2015a). Total RNA was isolated by the Siteman Cancer Center Tissue Processing Core using QIAGEN RNeasy kits. Single-indexed RNA sequencing (RNA-seq) libraries were prepared using the Illumina TruSeq Stranded Total RNA kit with 500 ng of starting material according to the manufacturer’s recommendations. Sequencing was performed on either the Illumina HiSeq 2500 V4 1 TB platform (2 3 125 bp reads) or the Illumina HiSeq 4000 platform (2 3 150 bp reads).

### Removing Contaminant Mouse Reads from Xenograft Data

WGS, WES, and RNA-seq reads from xenografts were aligned competitively against the human reference genome (National Center for Biotechnology Information [NCBI] build 38, GRCh38) and the mouse reference genome (Genome Reference Consortium Mouse Build 38, mm10) using the Xenome (version 1.0.0) software in order to filter mouse reads from human reads (Conway et al., 2012). Subsequent somatic variant detection was performed on data excluding the mouse-mapped reads.

### Sequence Alignment and Somatic Event Detection

The Genome Modeling System (GMS) was used for all analysis, including the somatic variant detection and RNA-seq analysis (Griffith et al., 2015b). Briefly, WGS and WES data were processed through SpeedSeq version 0.1.0 (Chiang et al., 2015; Faust and Hall, 2014), which aligns reads using BWA-MEM version 0.7.10 (Li, 2013) to the human reference genome (NCBI build 38, GRCh38) and marks duplicates using SAMBLASTER version 0.1.22 (Faust and Hall, 2014). RNA reads were aligned to GRCh38 using TopHat version 2.0.8 (Trapnell et al., 2009). Somatic variants were predicted using several variant callers by comparing primary tumor or xenograft with matched normal pairs. SNVs and small indels were detected and annotated using the GMS transcript variant annotator against Ensembl version 74. See Supplemental Experimental Procedures for more details. All SNVs and indels were manually reviewed for removal of false positives according to standard procedures (Barnell et al., 2018). Somatic CNAs were detected by CopyCat version 0.1 (https://github.com/chrisamiller/copyCat), and structural variations were predicted using Manta version 0.29.6 (Chen et al., 2016). Tumor purity was estimated by the mode of minor allele frequencies in regions of loss of heterozygosity (LOH), as previously described (Anagnostou et al., 2017). SciClone was used to assess the clonality of mutations present in copy neutral and non-LOH regions (Miller et al., 2014).

### Gene Expression and Pathway Analysis

Gene expression levels were quantified using Cufflinks version 2.1.1(Trapnell et al., 2010) and HTSeq-count version 0.5.4p1 (Anders and Huber, 2010). Differential expression analysis was performed using the DESeq2 R package (Love et al., 2014) on gene raw counts generated using HTSeq, and gene expression pathway analysis was performed using the GAGE R package (Luo et al., 2009).

### Analysis of Published Expression Data and Random Forest Classification

The microarray probe-level intensity files (containing log_2_-transformed, normexp background-corrected, LOESS-normalized values) from Walter et al. (2013) (GEO: GSE39366; n = 138) were gene median-normalized. Gene expression data (FPKM [fragments per kilobase of transcript per million mapped reads]) from the TCGA HNSCC cohort (n = 277) were log_2_-transformed and gene median-normalized (Cancer Genome Atlas Network, 2015). The randomForest R package version 4.6–12 was used to build a classifier using 638 Ensembl gene identifiers previously used to define the four molecular subtypes of HNSCC and trained on the dataset of Walter et al. (2013) (GEO: GSE39366) on the basis of their previously reported molecular subtypes. This classifier was subsequently validated on the TCGA dataset and used to predict gene expression subtypes in the reported dataset (WUSM; n = 16). The infiltration of CAFs was interrogated by summarizing the expression of 412 CAF-associated genes within the three datasets (Walter et al., 2013, TCGA, and WUSM). For further details, see Supplemental Experimental Procedures.

### Statistical Methods

Clinicopathological comparisons were conducted using chi-square tests or one-way ANOVA as appropriate. All statistics and data visualization were performed in R version 3.3.2. using the ggplot2 R package (version 2.2.1) (Wickham, 2009) and GenVisR version 1.8.0 (Skidmore et al., 2016). Concordance of expression and CNA was determined using the Pearson correlation, and VAF distribution between PDXs and matched tumors was summarized using the coefficient of determination.

## DATA AND SOFTWARE AVAILABILITY

The accession number for the sequencing data reported in this paper is database of Genotypes and Phenotypes (dbGaP): phs001623.v1.p1.

## Supplementary Material

Document S1

Table S2

Table S3

Table S4

## Figures and Tables

**Table 1.** Clinical Summary of Patient Samples

	Repository (n = 63)	Sequenced Subset (n = 16)
Standard-of-care resection	34 (54%)	6 (38%)
Trametinib trial	22 (35%)	10 (63%)
Pembrolizumab trial	7 (11%)	-
Treatment-naive	45 (71%)	14 (88%)
Post-treatment	15 (24%)	-
Relapse	3 (5%)	2 (13%)
I	3 (5%)	-
II	2 (3%)	-
III	12 (19%)	3 (19%)
IV	45 (71%)	13 (81%)
Below 40	4 (6%)	2 (13%)
40–59	22 (35%)	6 (38%)
60–79	29 (46%)	7 (44%)
Over 80	8 (13%)	1 (6%)
Male	46 (73%)	12 (75%)
Female	17 (27%)	4 (25%)

Overall, 63 PDXs were generated from 53 patients. In some cases, multiple PDXs were derived from the same patient at various time points. Numbers (n) reflect number of xenografts associated. Refer to Table S1 for additional information

## Highlights

- PDXs retain the genomic and transcriptomic profiles of matched primary OCSCC tumors
- Engraftment of PDXs is successful across expression and genomic subtypes of OCSCC
- PDXs generated in a “co-clinical trial” setting parallel patient trajectory
- PDXs serve as a translational platform for mechanistic drug discovery in OCSCC
