# Supplementary material for: Oral Cavity Squamous Cell Carcinoma Xenografts Retain Complex Genotypes and Intertumor Molecular Heterogeneity
Source: Cell Rep. Author manuscript; Available in PMC 2019 Mar 14. (PMC6417872; doi:10.1016/j.celrep.2018.07.058)
Supplement: Document S1 [file NIHMS1520774-supplement-Document_S1.pdf]

Cell Reports, Volume *24*

Supplemental Information

Oral Cavity Squamous Cell Carcinoma Xenografts

Retain Complex Genotypes

and Intertumor Molecular Heterogeneity

Katie M. Campbell, Tianxiang Lin, Paul Zolkind, Erica K. Barnell, Zachary L. Skidmore, Ashley E. Winkler, Jonathan H. Law, Elaine R. Mardis, Lukas D. Wartman, Douglas R. Adkins, Rebecca D. Chernock, Malachi Griffith, Ravindra Uppaluri, and Obi L. Griffith

SUPPLEMENTAL FIGURES

Figure S1. Targeted sequencing methods exhibit lower levels of mouse contamination. Refer to Experimental Procedures. A-B. Bar charts displaying either the raw number or proportion of reads classified by Xenome as confidently 'Human' or 'Mouse,' or as 'Both', 'Neither', or 'Ambiguous' for WGS (n=9), WES (n=16), or RNAseq (n=16) PDX samples. **C-D.** Distribution of the raw number or proportion of reads in each data type associated with each Xenome class.

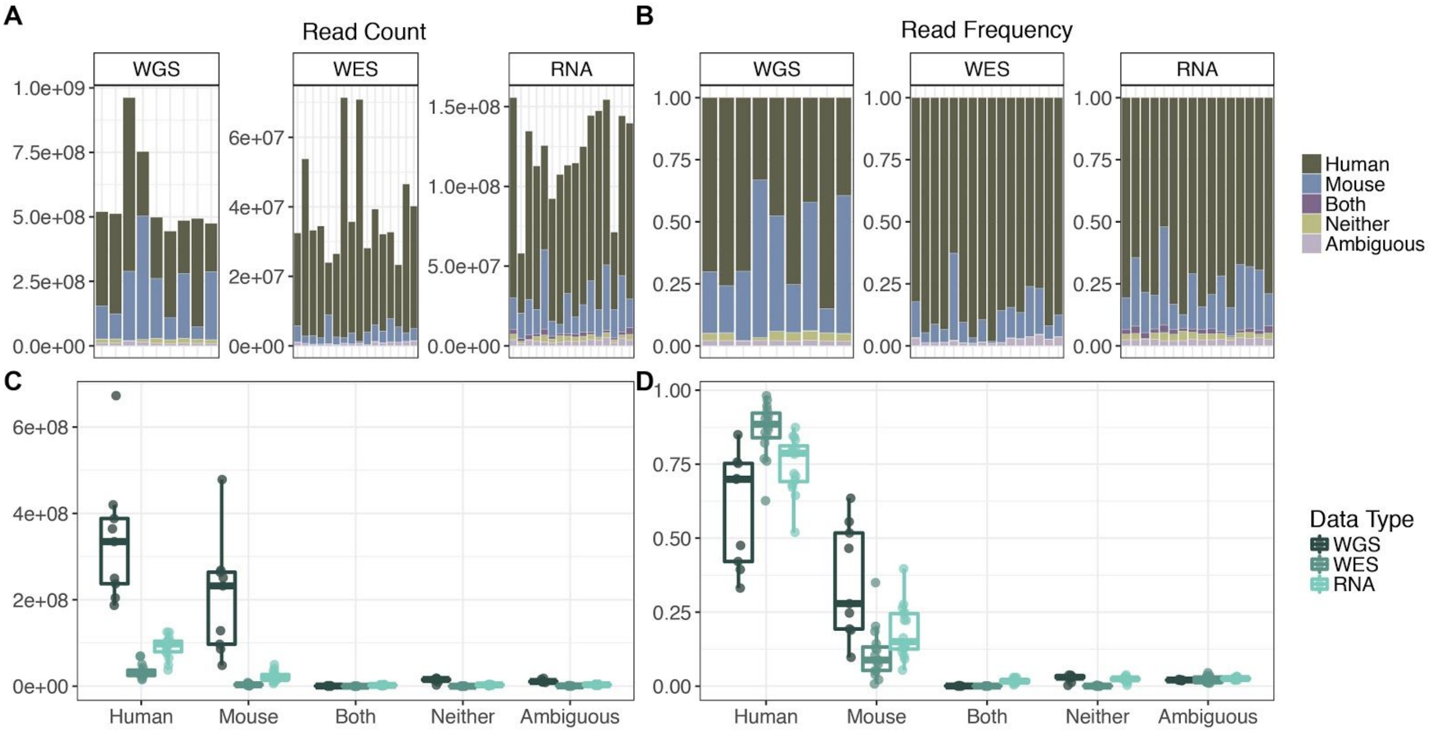

Figure S2. Somatic landscape across the sequenced PDX cohort. Refer to Figure 1. A. This waterfall plot shows recurrently mutated genes across the cohort of primary tumors and paired xenografts. The bar chart across the top shows the total number of nonsilent mutations identified across each case-matched PDX/tumor. Bars are filled to indicate the number of mutations detected in both tumor and PDX ('Common') or only one sample ('Tumor Only' or 'PDX Only'). The waterfall plot indicates the type of mutation detected in each gene (y-axis) for each sample (x-axis). Borders around tiles indicate whether the mutation was only detected in either the tumor or PDX ('Tumor Only' or 'PDX Only'). The horizontal bar chart on the left indicates the percentage of the cohort containing mutations in the indicated gene. **B.** Median absolute copy number was calculated across large chromosomal segments, and large copy number alterations were called across the genome. Colored tiles indicate the patient-associated samples. Within each row, the tumor is on the top and the xenograft is on the bottom. **C.** Genes commonly altered at the copy number level in HNSCC were analyzed with 100kb windows on either ends of the gene. Red rectangles correspond to the genomic positions of the indicated gene. Point color corresponds to sample, and copy neutral samples are indicated. Copy number is indicated by absolute copy number on the y-axis, and only segments with median copy number >3 or <1.5 are indicated by color (according to sample source). The horizontal dotted line at y=2 indicates copy neutral status.

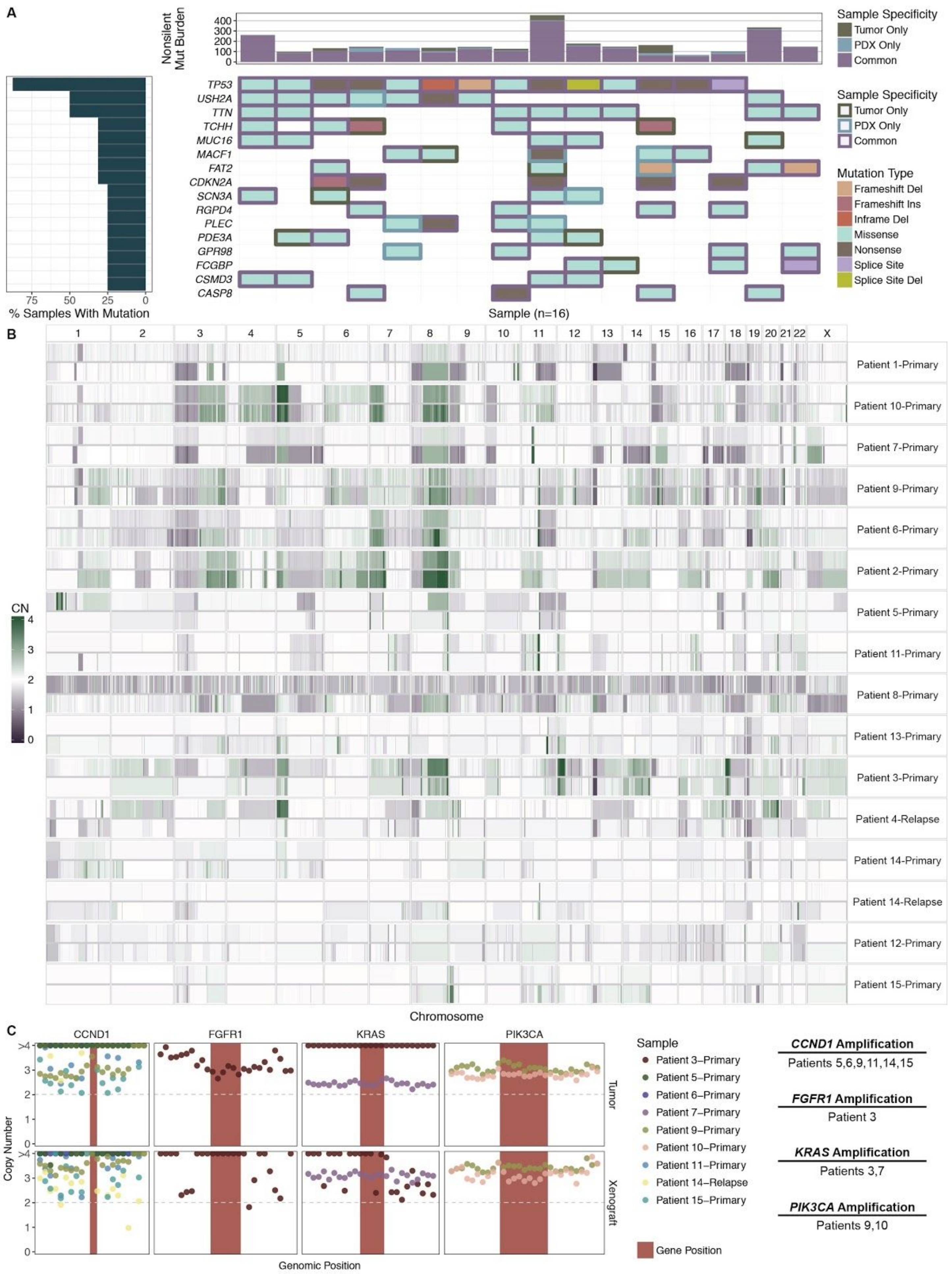

A
Nonsilent Mut Burden
Sample Specificity
Tumor Only
PDX Only
Common
TP53
USH2A
TTN
TCHH
MUC16
MACF1
FAT2
CDKN2A
SCN3A
RGPD4
PLEC
PDE3A
GPR98
FCGBP
CSMD3
CASP8
Mutation Type
Frameshift Del
Frameshift Ins
Inframe Del
Missense
Nonsense
Splice Site
Splice Site Del
% Samples With Mutation
Sample (n=16)
B
CN
Patient 1-Primary
Patient 10-Primary
Patient 7-Primary
Patient 9-Primary
Patient 6-Primary
Patient 2-Primary
Patient 5-Primary
Patient 11-Primary
Patient 8-Primary
Patient 13-Primary
Patient 3-Primary
Patient 4-Relapse
Patient 14-Primary
Patient 14-Relapse
Patient 12-Primary
Patient 15-Primary
Chromosome
C
CCND1
FGFR1
KRAS
PIK3CA
Tumor
Xenograft
Copy Number
Genomic Position
Sample
Patient 3–Primary
Patient 5–Primary
Patient 6–Primary
Patient 7–Primary
Patient 9–Primary
Patient 10–Primary
Patient 11–Primary
Patient 14–Relapse
Patient 15–Primary
Gene Position
CCND1 Amplification
Patients 5,6,9,11,14,15
FGFR1 Amplification
Patient 3
KRAS Amplification
Patients 3,7
PIK3CA Amplification
Patients 9,10

SUPPLEMENTAL TABLES

Table S1. Overview of the reported PDX repository. Refer to Table 1. *X = xenograft/standard-of-care resection, T = trametinib clinical cohort, P = pembrolizumab clinical trial cohort.

Cohort*	Patient	Timepoint	Treatment	TNM	Stage	Age (yrs)	Sex	Days to Harvest	Sequencing
T	1	primary	untreated	T4aN0M0	IVA	74	M	105	WES,RNA
T	1	posttreatment	trametinib	NA	NA	74	M	65	None
T	2	primary	untreated	T3N1M0	III	30	F	132	WGS,WES,RNA
T	2	posttreatment	trametinib	T1N0M0	I	30	F	36	WES
T	3	primary	untreated	T4aN1M0	IVa	45	M	85	WGS,WES,RNA
T	3	posttreatment	trametinib	T4aN0M0	IVA	45	M	101	None
X	4	relapse	post-surgery	T4bN2bMx	IVB	55	F	111	WGS,WES,RNA
X	5	primary	untreated	T4aN1Mx	IVA	84	F	88	WGS,WES,RNA
T	6	primary	untreated	T4aN2bM0	IV	65	M	117	WGS,WES,RNA
T	6	posttreatment	trametinib	T4aN2bM0	IV	65	M	95	None
X	7	primary	untreated	T2N1Mx	III	56	F	93	WGS,WES,RNA
X	8	primary	untreated	T3N2Mx	IVA	18	M	90	WGS,WES,RNA
X	9	primary	untreated	T2N1MX	III	69	M	85	WGS,WES,RNA
X	10	primary	untreated	T4aN2Mx	IVA	47	M	38	WGS,WES,RNA
T	11	primary	untreated	T3N2bM0	IVa	54	M	68	WES,RNA
T	12	primary	untreated	T2N2bM0	IVA	78	M	44	WES,RNA
T	12	posttreatment	trametinib	T1N1M0	III	78	M	27	None
T	13	primary	untreated	T4N2M0	IVA	57	M	115	WES,RNA
T	14	primary	untreated	T2N2bM0	IVA	75	M	247	WES,RNA
T	14	posttreatment	trametinib	T1N2bM0	IVA	75	M	203	None
T	14	relapse	post-surgery	rT4aN0	IVA	75	M	43	WES,RNA
T	15	primary	untreated	T4N0M0	IVA	66	M	84	WES,RNA
T	15	posttreatment	trametinib	T2N0M0	II	66	M	69	None
X	16	primary	untreated	T4N2bM0	IVA	71	M	84	None
X	17	primary	untreated	rT4aN0Mx	IVA	81	F	44	None
X	18	relapse	post-surgery	T4AN1Mx	IVA	69	M	137	None
X	19	primary	untreated	T4aN2Mx	IVA	47	M	78	None
X	20	primary	untreated	T4aN1Mx	IVA	60	M	83	None
X	21	primary	untreated	T4aN2Mx	IVA	58	M	57	None
X	22	primary	untreated	T3N0Mx	III	54	M	38	None
X	23	primary	untreated	T4aN2Mx	IVA	50	M	59	None
X	24	primary	untreated	T4aN0Mx	IVA	64	M	81	None
X	25	primary	untreated	T4aN0Mx	IVA	39	M	165	None
X	26	primary	untreated	pT3NxMx	III	86	F	106	None
X	27	primary	untreated	T1N0Mx	I	60	M	217	None
X	28	primary	untreated	T3N1Mx	III	67	M	68	None
X	29	primary	untreated	T2M0Mx	III	43	M	111	None
X	30	primary	untreated	T3N2Mx	IVA	53	M	124	None
X	31	primary	untreated	rT4N1M0	IVA	85	M	133	None
X	32	primary	untreated	T1N0Mx	I	61	M	98	None
X	33	primary	untreated	T2N2Mx	IVA	51	F	66	None

X	34	primary	untreated	T4aN0Mx	IVA	55	F	85	None
X	35	primary	untreated	T4aNxM0	IVA	80	M	96	None
X	36	primary	untreated	T4aNxMx	IVA	58	F	76	None
X	37	primary	untreated	pT4NxMx	IVA	83	F	NA	None
X	38	primary	untreated	T3N0Mx	III	54	M	NA	None
X	39	primary	untreated	T2N0	II	49	F	117	None
X	40	primary	untreated	T3N2Mx	IVA	71	F	NA	None
X	41	primary	untreated	T4aN1Mx	IVA	54	M	42	None
X	42	primary	untreated	T3N1Mx	III	79	F	NA	None
X	43	primary	untreated	T4aN2bM1	IVC	64	F	76	None
T	44	primary	untreated	T2N1M0	III	58	M	217	None
T	45	primary	untreated	T4aN2CM0	IVa	63	M	281	None
T	46	posttreatment	trametinib	T4aN0M0	IVA	63	M	82	None
T	47	posttreatment	trametinib	T1N2bM0	III	59	M	65	None
T	48	posttreatment	trametinib	T4aN2bM0	IVa	72	M	143	None
P	49	posttreatment	pembrolizumab	T4N2C	IV	60	M	57	None
P	50	primary	untreated	T4N2B	IV	87	M	110	None
P	51	posttreatment	pembrolizumab	T4N2B	IV	87	M	42	None
P	52	posttreatment	pembrolizumab	T4N0	IV	72	F	50	None
P	53	primary	untreated	T4N0	IV	73	M	98	None
P	54	posttreatment	pembrolizumab	T4N1	IV	54	M	47	None
P	55	posttreatment	pembrolizumab	T4N1	IV	69	F	44	None

SUPPLEMENTAL EXPERIMENTAL PROCEDURES

Xenoengraftment procedures

Tumor biopsies were obtained from patients and and maintained in sterile Dulbecco Modified Eagle's medium containing 10% Fetal Calf Serum (FCS) and 1% amphotericin. Biopsies were sectioned using razor blades into four separate pieces for the following purposes: (1) formalin fixation for storage and immunohistochemical analysis (2) flash freezing in liquid nitrogen for DNA and RNA extraction, (3) slow freezing in FCS containing 10% dimethyl sulfoxide (DMSO) for downstream experiments, and (4) immediate transplantation into mice for xenograft generation.

Within one hour of acquisition, fresh tumor was immediately minced into approximately 16 pieces, ranging in size from 2-8 mm3, and transferred on ice to the animal facility. 6-8 week old NOD-scid ILRgnull (NSG) mice (Jackson Laboratory, Bar Harbor, ME) were anesthetized, shaved, and four small incisions were made, one on each quadrant of the flank. Tumor pieces were then saturated with Matrigel (Corning, Tewksbury, MA), and four pieces were transferred subcutaneously into each quadrant using sterile forceps.

Mice were maintained on sterile water containing sulfamethoxazole (280 ug/mL) and trimethoprim (56 ug/mL) for one month after injection and monitored twice weekly for tumor growth. Successful establishment was defined as progressive tumor growth and tumors were harvested at approximately 2 cm3 tumor volume. Xenografts were resected and divided in the same manner as the primary tumors and were named the "P0" generation. In some cases, P0 generation tumors were slow-frozen in FCS+10% DMSO and thawed for subsequent engraftment of the P1 generation.

Trametinib treatment of engrafted mice

Trametinib (Selleckchem, Houston, TX) was dissolved in DMSO (10 mg/mL), and further diluted into sterile water containing 0.5% w/v hypromellose (Sigma-Aldrich, St. Louis, MO) and 2% v/v Tween-80 (Sigma-Aldrich, St. Louis, MO) to a concentration of 0.3 mg/mL (Banks et al., 2015). Mice bearing successfully engrafted OCSCC tumors were treated with daily oral gavage with either trametinib (3 mg/kg), or vehicle alone beginning 7 days after implantation. Tumor dimensions were measured daily.

Sequencing methods

Library construction and sequencing were performed as previously described with a few exceptions described below(Griffith et al., 2015a). Single indexed libraries were constructed according to the manufacturer's recommendations using the Illumina TruSeq Nano Kit (Illumina Inc, San Diego, CA) for whole genome sequencing (WGS) on the Illumina HiSeq X (2x150 bp reads). Genomic DNA was fragmented using the Covaris E210 DNA Sonicator (Covaris, WoBurn, MA). Dual indexed whole exome sequencing (WES)

libraries were constructed/pooled according to the manufacturer's recommendations using one of three kits/approaches: (1) the Paired-End Sample Prep Kit (Illumina Inc, San Diego, CA) for sequencing on the HiSeq 2500 (2x125 bp reads) (2) Kapa Auto Illumina (Kapa Biosystems, Woburn, MA) for sequencing on the HiSeq 2500 V4 1Tb (2x125 bp reads) and (3) Kapa Auto Illumina (Kapa Biosystems, Woburn, MA) for sequencing on the HiSeq 4000 (2x150 bp reads). Samples were pooled and captured using one of four capture reagents: (1) NimbleGen SeqCap EZ Human Exome Library v3.0 Kit (Roche NimbleGen, Madison, WI) (2) NimbleGen SeqCap EZ Human Exome Library v3.0 Kit spiked with a custom capture Integrated DNA technologies (IDT) reagent (Griffith et al., 2015a) (3) NimbleGen SeqCap EZ HGSC VCRome Kit (Roche NimbleGen, Madison, WI) and (4) xGen Lockdown Exome Panel v1.0 (IDT, Coralville, IA).

Somatic event detection
The Genome Modeling System (GMS) was used for all analysis, including the somatic variant detection and RNA-seq analysis (Griffith et al., 2015b). Single nucleotide variants (SNVs) were detected by taking the union of VarScan2 v2.3.6(Koboldt et al., 2012), Strelka v1.0.11(Saunders et al., 2012), Mutect v1.1.4(Cibulskis et al., 2013), and SomaticSniper v1.0.4(Larson et al., 2012), and filtered using Samtools r982(Li et al., 2009) ([mpileup -BuDS] filtered by var-filter-snv v1 then false-positive-vcf v1). Small insertions and deletions (indels) were detected by GATK Somatic Indel Detector (v5336) (McKenna et al., 2010), VarScan2, Strelka, and Mutect. Variants were annotated by the GMS transcript variant annotator against Ensembl v74 and compared to the database of curated mutations (DoCM) (Ainscough et al., 2016; Chen et al., 2016) and COSMIC mutations (Forbes et al., 2011). All SNVs and indels were manually reviewed for removal of false positives according to standard procedures (Barnell et al., 2018).

Analysis of published expression data and random forest classification.
728 genes were previously used to define four molecular subtypes of HNSCC using this gene expression dataset (Walter et al., 2013). These 728 genes (HGNC symbols) mapped to 797 gene identifiers in the Ensembl v90 database. The union of Ensembl gene identifiers was taken across three experiments - Walter et al., the TCGA HNSCC dataset (Cancer Genome Atlas Network, 2015), and this study (hereafter referred to as WUSM) - to produce a final list of 638 Ensembl gene IDs. The microarray probe-level intensity files (containing log2-transformed, normexp background-corrected, loess-normalized values) from Walter et al. (GSE39366, n=138) were gene median-normalized (Walter et al., 2013). Gene expression data (FPKM) from the TCGA HNSCC cohort (n=277) was log2-transformed and gene median-normalized (Cancer Genome Atlas Network, 2015). The randomForest R package v.4.6-12 was used to build a classifier using the 638 genes and the 138 samples from the Walter et al. dataset (GSE39366) based upon their previously reported molecular subtypes . The classifier was defined using 1,001 trees and downsampling to the minimum sample size per molecular subtype (n=29). Model performance was validated using the randomForest package by applying the classifier to the TCGA dataset and comparing predictions to the previously reported molecular subtypes. Tumor RNA expression (FPKM) reported in this study (WUSM; n=16) was log2-transformed and gene median-normalized, and molecular subtypes were predicted by applying the classifier to these expression values.

Genes associated with cancer-associated fibroblasts (CAFs) were used to compare classified mesenchymal tumors with others, based upon the transformed/gene median-normalized expression values within each dataset (Walter et al., TCGA, WUSM). Puram et al. showed that expression of 449 genes can be used to describe a signature associated with cancer-associated fibroblasts (CAFs) that defines the mesenchymal molecular subtype of head and neck cancers (Puram et al., 2017). These 449 genes mapped to 412 Ensembl gene identifiers assessed in the Walter et al., TCGA, and WUSM datasets. The 412 genes associated with CAF expression signatures were gene-median centered (GMC) with respect to each dataset, and then these 412 genes were summarized per sample by the median GMC value (denoted as 'CAF signature' in Figure 5).

SUPPLEMENTAL REFERENCES

Ainscough, B.J., Griffith, M., Coffman, A.C., Wagner, A.H., Kunisaki, J., Choudhary, M.N., McMichael, J.F., Fulton, R.S., Wilson, R.K., Griffith, O.L., et al. (2016). DoCM: a database of curated mutations in cancer. Nat. Methods *13*, 806–807.

Barnell, E.K., Ronning, P., Campbell, K.M., Krysiak, K., Ainscough, B.J., Ramirez, C., Spies, N., Kunisaki, J., Hundal, J., Skidmore, Z.L., et al. (2018). Standard operating procedure for somatic variant refinement of tumor sequencing data.

Cancer Genome Atlas Network (2015). Comprehensive genomic characterization of head and neck squamous cell carcinomas. Nature *517*, 576–582.

Chen, X., Schulz-Trieglaff, O., Shaw, R., Barnes, B., Schlesinger, F., Källberg, M., Cox, A.J., Kruglyak, S., and Saunders, C.T. (2016). Manta: rapid detection of structural variants and indels for germline and cancer sequencing applications. Bioinformatics *32*, 1220–1222.

Cibulskis, K., Lawrence, M.S., Carter, S.L., Sivachenko, A., Jaffe, D., Sougnez, C., Gabriel, S., Meyerson, M., Lander, E.S., and Getz, G. (2013). Sensitive detection of somatic point mutations in impure and heterogeneous cancer samples. Nat. Biotechnol. *31*, 213–219.

Forbes, S.A., Bindal, N., Bamford, S., Cole, C., Kok, C.Y., Beare, D., Jia, M., Shepherd, R., Leung, K., Menzies, A., et al. (2011). COSMIC: mining complete cancer genomes in the Catalogue of Somatic Mutations in Cancer. Nucleic Acids Res. *39*, D945–D950.

Griffith, M., Miller, C.A., Griffith, O.L., Krysiak, K., Skidmore, Z.L., Ramu, A., Walker, J.R., Dang, H.X., Trani, L., Larson, D.E., et al. (2015a). Optimizing cancer genome sequencing and analysis. Cell Syst *1*, 210–223.

Griffith, M., Griffith, O.L., Smith, S.M., Ramu, A., Callaway, M.B., Brummett, A.M., Kiwala, M.J., Coffman, A.C., Regier, A.A., Oberkfell, B.J., et al. (2015b). Genome Modeling System: A Knowledge Management Platform for Genomics. PLoS Comput. Biol. *11*, e1004274.

Koboldt, D.C., Zhang, Q., Larson, D.E., Shen, D., McLellan, M.D., Lin, L., Miller, C.A., Mardis, E.R., Ding, L., and Wilson, R.K. (2012). VarScan 2: somatic mutation and copy number alteration discovery in cancer by exome sequencing. Genome Res. *22*, 568–576.

Larson, D.E., Harris, C.C., Chen, K., Koboldt, D.C., Abbott, T.E., Dooling, D.J., Ley, T.J., Mardis, E.R., Wilson, R.K., and Ding, L. (2012). SomaticSniper: identification of somatic point mutations in whole genome sequencing data. Bioinformatics *28*, 311–317.

Li, H., Handsaker, B., Wysoker, A., Fennell, T., Ruan, J., Homer, N., Marth, G., Abecasis, G., Durbin, R., and 1000 Genome Project Data Processing Subgroup (2009). The Sequence Alignment/Map format and SAMtools. Bioinformatics *25*, 2078–2079.

McKenna, A., Hanna, M., Banks, E., Sivachenko, A., Cibulskis, K., Kernytsky, A., Garimella, K., Altshuler, D., Gabriel, S., Daly, M., et al. (2010). The Genome Analysis Toolkit: a MapReduce framework for analyzing next-generation DNA sequencing data. Genome Res. *20*, 1297–1303.

Puram, S.V., Tirosh, I., Parikh, A.S., Patel, A.P., Yizhak, K., Gillespie, S., Rodman, C., Luo, C.L., Mroz, E.A., Emerick, K.S., et al. (2017). Single-Cell Transcriptomic Analysis of Primary and Metastatic Tumor Ecosystems in Head and Neck Cancer. Cell *171*, 1611–1624.e24.

Saunders, C.T., Wong, W.S.W., Swamy, S., Becq, J., Murray, L.J., and Cheetham, R.K. (2012). Strelka: accurate somatic small-variant calling from sequenced tumor-normal sample pairs. Bioinformatics *28*, 1811–1817.

Walter, V., Yin, X., Wilkerson, M.D., Cabanski, C.R., Zhao, N., Du, Y., Ang, M.K., Hayward, M.C., Salazar, A.H., Hoadley, K.A., et al. (2013). Molecular subtypes in head and neck cancer exhibit distinct patterns of chromosomal gain and loss of canonical cancer genes. PLoS One *8*, e56823.
